# Emerging Roles of Long Noncoding RNAs in Breast Cancer Epigenetics and Epitranscriptomics

**DOI:** 10.3389/fcell.2022.922351

**Published:** 2022-07-05

**Authors:** Elżbieta Wanowska, Klaudia Samorowska, Michał Wojciech Szcześniak

**Affiliations:** ^1^ Department of Biological Sciences, Auburn University, Auburn, AL, United States; ^2^ Institute of Human Biology and Evolution, Faculty of Biology, Adam Mickiewicz University in Poznan, Poznań, Poland

**Keywords:** breast cancer, epigenetics, epitranscriptomics, long noncoding RNAs, RNA modifications

## Abstract

Breast carcinogenesis is a multistep process that involves both genetic and epigenetic changes. Epigenetics refers to reversible changes in gene expression that are not accompanied by changes in gene sequence. In breast cancer (BC), dysregulated epigenetic changes, such as DNA methylation and histone modifications, are accompanied by epitranscriptomic changes, in particular adenine to inosine modifications within RNA molecules. Factors that trigger these phenomena are largely unknown, but there is evidence for widespread participation of long noncoding RNAs (lncRNAs) that already have been linked to virtually any aspect of BC biology, making them promising biomarkers and therapeutic targets in BC patients. Here, we provide a systematic review of known and possible roles of lncRNAs in epigenetic and epitranscriptomic processes, along with methods and tools to study them, followed by a brief overview of current challenges regarding the use of lncRNAs in medical applications.

## Introduction

**FIGURE 1:**
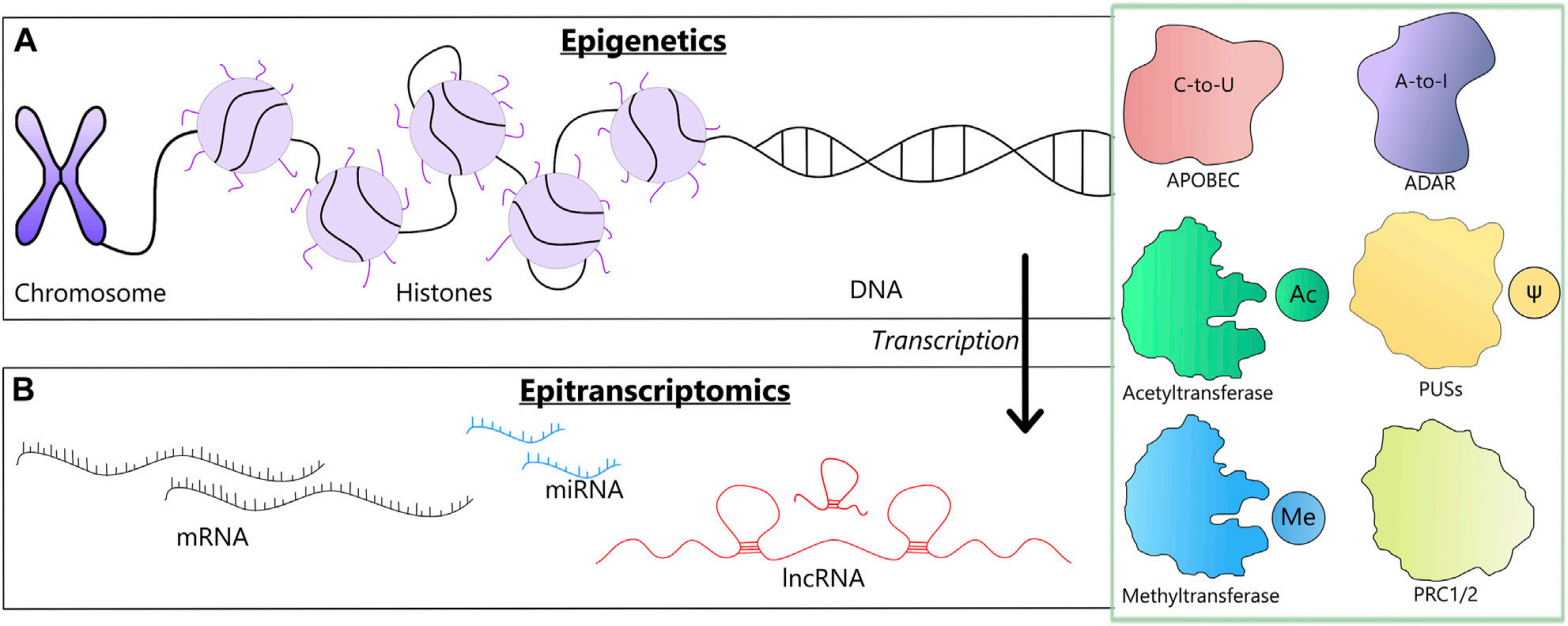
A schematic representation of two levels at which the reviewed modifications may occur along with the involved enzymes (a green box). **(A)** Epigenetic changes could involve chromosome remodeling, histone modifications and DNA methylation. **(B)** Epitranscriptomic changes, involve changes in mRNAs, tRNAs and lncRNAs, such as RNA editing, m6A modification and pseudouridilation.

According to the Global Cancer Observatory (https://gco.iarc.fr/), female breast cancer (BC) ranks as the second most diagnosed cancer and the fifth leading cause of cancer-related deaths worldwide. This fact exists despite the etiology of BC being subject to extensive research, which has led to significant advances in diagnostic and prognostic approaches as well as BC treatment, ranging from surgery through chemotherapy to hormonal, biological, or radiation therapies (https://www.cdc.gov). One of the reasons is that BC is a complex disease, with its etiology involving both environmental and hereditary factors; it is also highly heterogeneous, as it is typically classified into six molecular subtypes: luminal A, luminal B, normal-like, basal (also known as triple-negative), HER+, and claudin-low based on their unique phenotypes and molecular characteristics, such as hormone receptors and human EGF-like receptor 2 (HER2) receptor status (Dai et al., 2017). Importantly, different subtypes have distinct clinical outcomes and therapeutic responses. Elucidating novel BC-related molecular mechanisms is critical for improving its treatment, with ongoing research occurring in diverse directions. Notably, many studies have revealed a strong association of long noncoding RNAs (lncRNAs) with BC etiology (Jin et al., 2021; Selem et al., 2021; Cava et al., 2022; Zhu and Zhu, 2022). LncRNAs are defined as transcripts that are not translated into functional proteins and are at least 200 nt long. They might be able, however, to encode micropeptides (Jin et al., 2021; Polenkowski et al., 2022; Setrerrahmane et al., 2022), which are shorter than 100 amino acids but typically much shorter than that. Micropeptides originating from lncRNAs may play ambiguous roles in cancer development, as they are known to either promote or suppress the tumor (Ye et al., 2020). For instance, in triple-negative breast cancer a small peptide encoded by LINC00908 has antitumor properties and is believed to represent a promising target for cancer treatment (Wang et al., 2019). LncRNAs have important documented cellular functions, as recently reviewed (Statello et al., 2021), and they have also been implicated in virtually any hallmark of cancer cells, from their capacity for proliferation and survival through increased metabolism to their relationship with the tumor microenvironment. Examples include lncRNAs involved in transcriptional regulation by key oncogenic or tumor-suppressive transcription factors, such as p53 (Sanchez et al., 2014) or MYC (Hart et al., 2014), with a number of instances related to BC (Dvorska et al., 2021; Wang et al., 2021). Of note, many lncRNAs are linked to epigenetic processes within a cell. Epigenetic modifications, such as histone modifications, DNA methylation, or nucleosome remodeling (Figure 1A) as well as epitranscriptomic changes such as RNA editing, m6A modification and pseudouridilation (Figure 1B) are of particular interest because they play a role in oncogene overexpression or tumor suppressor gene silencing, thereby stimulating tumorigenic pathways and influencing therapeutics in BC (Sher et al., 2020; Zhao C. et al., 2021). They are also known to modulate a variety of molecular, cellular, and biological pathways involved in breast carcinogenesis and play roles in cancer hallmarks such as drug resistance and stemness (Pasculli et al., 2018). Some of the changes affect cell function and contribute to oncogenic transformation. By blocking or reversing these modifications with drugs or gene therapy, the cancer phenotype could be restored to normal. Thus, some lncRNAs with functions related to epigenetic processes represent promising therapeutic targets, with a variety of strategies being recommended, including suppression of oncogenic lncRNAs, altering their epigenetic effects, interfering with their function, restoring downregulated or lost lncRNAs, and recruiting lncRNA regulatory elements. In this review, we aim to cover the involvement of lncRNAs in epigenetic and epitranscriptomic changes that contribute to BC pathogenesis, including the genetic reprogramming of oncogenes and tumor suppressor genes (Table 1). This is followed by an overview of low- and high-throughput assays serving to study the lncRNA’s roles in BC epigenetics. Finally, we discuss the advantages and disadvantages as well as progress in the application of lncRNAs as epigenetic biomarkers in diagnostics and targets for BC therapeutics.

**TABLE 1:** A summary for lncRNAs with roles in breast cancer epigenetics and epitranscriptomics.

LncRNA name	Role in BC	Molecular mechanism	References
RUNXOR	tumor suppressor	Triggers DNA demethylation, H3K4me3 and activates expression of *RUNX1*	Nie et al. (2019)
H19	drug resistance	Mediates DNA methylation of *NAT1*	Sun et al. (2022)
91H	oncogenic	Prevents methylation at *H19/IGF2* locus	Vennin et al. (2017)
EPB41L4A-AS2	tumor suppressor	Regulated by ZNF217—mediated H3K27me3, increases *RARRES1* expression	Pang et al. (2010)
BLAT1	oncogenic	Regulated *via* DNA methylation at CpG islands within its promoter	Han et al. (2018)
HUMT	oncogenic	Regulated by promoter hypomethylation, modulates FOXK1 expression by recruiting YBX1	Zheng et al. (2020)
LINC02273	oncogenic	Mediates H3K4me3 and enhances AGR2 expression	Xiu et al. (2019)
PHACTR2-AS1	tumor suppressor	Induces H3K9me of rDNA	Chu et al. (2020)
AGAP2-AS1	drug resistance	Modulates H3K27ac and leads to upregulation of MyD88	Dong et al. (2018)
TINCR	drug resistance	Undergoes H3K27 acetylation, leading to its enhanced transcription	Dong et al. (2019)
HOTAIR	oncogenic	Promotes H3K28me3 genome-wide	Gupta et al. (2010)
PVT1	oncogenic	Inhibits the expression of *FOXF1* by recruiting EZH2	Guo et al. (2018)
ANCR	tumor suppressor	Affects EZH2 stability	Li Z. et al. (2017)
HIF1A-AS2	oncogenic	Undergoes ADAR1- dependent A-to-I editing	Ma et al. (2019)
LINC00944	oncogenic	Possibly regulated by ADAR’s interactions with Dicer or Staufen proteins	de Sentiago et al. (2021)
DLGAP1-AS1	drug resistance	Upregulated by WTAP, acts as miR-299-3p sponge	Huang et al. (2021)
LINC00958	oncogenic	Upregulated by m6A modification, acts as a ceRNA for miR-378a-3p	Rong et al. (2021)
LINC00675	tumor suppressor	Undergoes m6A modification, acts as ceRNA for miR-513b-5p	Fan and Wang, (2021)
MALAT1	oncogenic	Undergoes m6A modification, acts as a decoy for miRNA miR-26b	Zhao C. et al. (2021)

### DNA Methylation

**FIGURE 2:**
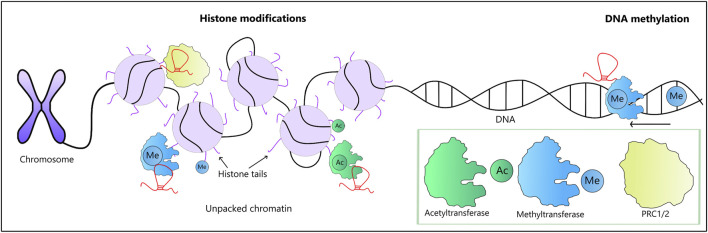
LncRNA-mediated epigenetic modifications along with the involved enzymes (a green box). Chromatin remodeling may be affected by lncRNAs interacting with remodeling enzymes, such as PRC1 and PRC2. These modifications either enhance or suppress the activity of involved genes. Two types of histone modifications are shown here: histone methylation carried out by methyltransferases (in blue) and acetylation carried out by acetyltransferases (in green), both of which are subject to modulation by lncRNAs. Of note, lncRNAs involved in DNA methylation may either enhance or suppress the process.

DNA methylation is an epigenetic mechanism during which a methyl group is added to the C5 position of the cytosine, resulting in the formation of 5-methylcytosine (Figure 2). Several epigenome-wide studies have revealed that changes in DNA methylation are associated with a higher risk of breast cancer (Xu et al., 2013; van Veldhoven et al., 2015; Xu et al., 2020), and a growing body of evidence indicates that lncRNAs are essential players in this process. Some of them modulate the expression of oncogenes or tumor suppressors by altering their methylation, such as *RUNX1* overlapping RNA (RUNXOR). RUNXOR triggers DNA demethylation, activating the expression of *RUNX1*, which appears to function in a context-dependent manner, with evidence supporting both tumor suppressor and oncogenic functions (Janes, 2011; Wang et al., 2011; Ferrari et al., 2014; Chimge et al., 2016; Nie et al., 2019). LncRNAs can influence not only tumorigenesis but also control drug resistance in human malignancies. One of the most prominent examples is a multifunctional lncRNA dubbed H19, which has been demonstrated to alter the DNA methylation associated with the development of BC (Zhou et al., 2015). Recently, Sun et al. has linked H19-mediated methylation of the *N-acetyltransferase 1* (*NAT1*) promoter to resistance to hormone therapy (Sun et al., 2022). Interestingly, H19 has its antisense transcript dubbed 91H, which increases the aggressive phenotype of BC cells by preventing DNA methylation at the H19/IGF2 locus (Vennin et al., 2017).

In addition to mediating DNA methylation, emerging evidence shows that lncRNA genes can also be methylated. Zhao et al. analyzed the epigenetic changes of lncRNAs in three breast cancer subtypes and identified three DNA methylation-dysregulated lncRNAs (CTC-303L1.2, RP11-738B7.1, and SLC26A4-AS1) as prognostic biomarkers of the basal subtype (Zhao et al., 2021). They discovered that DNA hypermethylation of CTC-303L1.2 enhancer regions results in decreased expression, which is associated with poorer patient survival. On the other hand, hypermethylation of the SLC26A4-AS1 promoter causes upregulation of SLC26A4-AS1, which is linked to longer overall survival. Finally, hypomethylation at the enhancer of RP11-738B7.1 causes its upregulation, but this is associated with a poor outcome. Another example of methylated lncRNA is EPB41L4A-AS2, which impedes the proliferation, migration, and invasion of BC cells. In addition to DNA methylation, EPB41L4A-AS2 is also regulated by ZNF217 (zinc finger protein 217)-mediated H3K27me3 (trimethylation of lysine 27 on histone 3), which contributes to inhibited expression of this lncRNA, thus limiting its tumor suppressor activity (Pang et al., 2019). Two other lncRNAs, *Basal-Like breast cancer Associated Transcript 1* (BLAT1) and *highly upregulated in metastatic TNBC* (HUMT), are epigenetically regulated in BC, but in contrast to EPB41L4A-AS2, they exhibit pro-oncogenic properties. Their hypomethylation leads to enhanced transcription, contributing to aggressive clinical features (Han et al., 2018; Zheng et al., 2020).

### Histone Modifications

Histone modifications play essential roles in the tumorigenesis and progression of BC, with changes in their patterns having a variety of effects (Elsheikh et al., 2009). One of them is histone methylation, a post-translational modification of lysine and arginine residues (Figure 2). RUNXOR, a lncRNA mentioned earlier, has been linked to the formation of histone H3K4me3 (trimethylation on histone H3 lysine 4) epigenetic marks in the *RUNX1* promoter in BC cells. Using chromatin immunoprecipitation qPCR (ChIP–qPCR), Nie et al. showed that the H3K4me3 mark is increased more than 20-fold in RUNXOR-overexpressing cells (Nie et al., 2019). The same type of modification is mediated by LINC02273, resulting in enhanced transcription of the AGR2 oncogene in BC metastasis. The expression level of LINC02273 is significantly elevated in metastatic lesions compared to primary tumors. Interestingly, this lncRNA is stabilized by heterogeneous nuclear ribonucleoprotein L (hnRNPL), which is also overexpressed in metastatic tissue (Xiu et al., 2019). In contrast to those cancer-promoting lncRNAs, PHACTR2-AS1 acts against tumor growth and metastasis in BC. A recent report showed that this lncRNA induces H3K9me (methylation of histone H3 at lysine 9) of ribosomal DNA, suppressing rRNA transcription. Chu et al. found that PHACTR2-AS1 is downregulated in BC patients and showed that lower expression of PHACTR2-AS1 promotes BC development and correlates with poor patient outcome (Chu et al., 2020).

The addition of an acetyl group to the lysine residues in histone tails, referred to as histone acetylation, constitutes another epigenetic mechanism in BC biology with the participation of lncRNAs. Changes in acetylation of histone H3 and H4 have been linked to tumor progression in different cancer types, with a global-scale alteration of histone acetylation during BC progression as well. For example, acetylation of histone H4 is markedly reduced in ductal carcinoma *in situ* and in invasive ductal carcinoma relative to normal breast epithelium (Suzuki et al., 2009). By affecting histone acetylation, lncRNAs facilitate access to DNA and activate gene expression. For instance, AGAP2-AS1 leads to upregulation of the carcinogenic protein MyD88 by modulating H3K27 acetylation (Dong et al., 2018), promoting chemoresistance of BC. It is worth noting that similar to DNA methylation, some lncRNAs are subject to histone modifications themselves, as is the case in TINCR, which undergoes H3K27 acetylation, leading to its enhanced transcription and, consequently, drug resistance (Dong et al., 2019).

### Interactions With Chromatin Remodeling Complexes

In addition to direct interactions with DNA and histones, lncRNAs have been demonstrated to participate in epigenetic regulation through interactions with chromatin-modifying enzymes, which catalyze covalent changes of histones or DNA to affect gene expression. For instance, KCNQ1OT1 that is overexpressed in BC mediates the H3K27me3 accumulation by recruiting histone methyltrasferases (Pandey et al., 2008; Andresini et al., 2019; Cagle et al., 2021). Polycomb repressive complexes 1 and 2 (PRC1 and PRC2) are multiprotein complexes with histone modification activity that frequently work together to repress gene expression (Figure 2). LncRNAs affect PRCs in a variety of ways. The expression of HOTAIR is elevated both in primary and metastatic tumors and promotes selective retargeting of PRC2 and H3K28me3 (trimethylation of histone H3 at lysine 28) genome-wide, increasing BC invasiveness and metastasis (Gupta et al., 2010). Another example is PVT1, which acts as an oncogenic lncRNA in cancers, including BC. Guo et al. showed that PVT1 inhibits the expression of the tumor suppressor gene *FOXF1* by recruiting EZH2, a catalytic subunit of the PRC2 complex, consequently, increasing H3K27me3 levels in BC (Guo et al., 2018). Last but not least, coming into interactions with chromatin-modifying enzymes not only serves scaffolding and recruitment functions but also may lead to degradation of the proteins and their complexes, as is the case in ANCR, which affects EZH2 stability and inhibits epithelial-mesenchymal transition (EMT), invasion, tumorigenesis and distant metastasis in BC (Li Z. et al., 2017).

### RNA Editing

**FIGURE 3:**
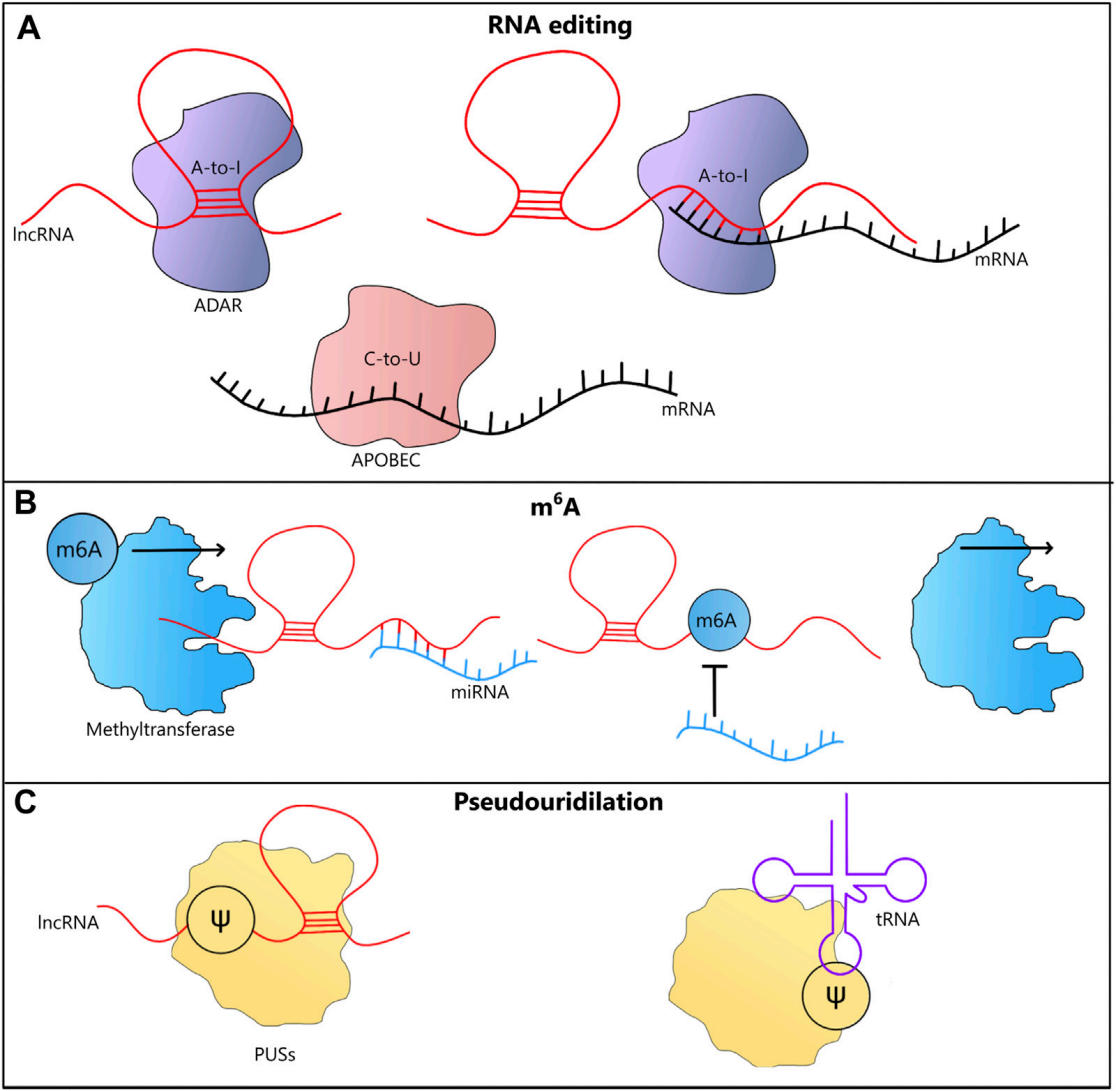
LncRNA-mediated epitranscriptomic modifications. **(A)** A-to-I RNA editing in lncRNAs, may be triggered by their ability to form dsRNA structures. LncRNAs may also trigger A-to-I RNA editing in mRNAs by coming into direct interactions with them. Both A-to-I and C-to-U RNA editing could affect the coding potential and therefore influence the functions played by proteins. **(B)** m6A modification stabilizes RNAs, which is often associated with their increased cellular levels. In case of lncRNAs that act as miRNA sponges, after this type of methylation their functions may be affected, leading to increased levels of miRNAs. **(C)** Pseudouridilation (Ѱ) conducted by PUSs enzymes alters interactions between bases and often leads to changes in the structure of lncRNA and tRNA molecules, affecting their functions.

The abovementioned epigenetic modifications represent a fraction of possibilities for lncRNAs to contribute to the complex biology of cancers, with epitranscriptomic modifications, such as RNA editing and m6A modification, emerging as an essential yet understudied area (Figure 3A). There are two major types of RNA editing, namely, adenosine-to-inosine (A-to-I) and cytosine-to-uracil (C-to-U) conversion (Romano et al., 2020). The former is carried out in double-stranded RNAs by adenosine deaminases acting on RNA (ADAR) enzymes ADAR1 and ADAR2 (Nishikura, 2016; Wang et al., 2021). Adenosines edited to inosines are read as guanosine during translation, which has essential functional implications. For instance, in mRNAs of the chloride channel Gabra3, an isoleucine is substituted with methionine in a subunit of the receptor GABAA. Interestingly, while overexpression of Gabra3 promotes BC cell migration, invasion, and metastasis, the edited Gabra3 has the opposite properties (Gumireddy et al., 2016). On the other hand, RNA editing in 3’ UTRs may disrupt the direct interaction of microRNAs (miRNAs) with mRNAs or RNA binding proteins, which often affects their expression levels or processing (Nishikura, 2016). In BC tumors, there is an increased level of editing at the 3’UTR sites of genes that are relevant to the metabolism of ncRNAs (noncoding RNAs) or the DNA damage response, as well as in genes involved in important signaling pathways, such as ATM, GINS4, or POLH (Sagredo et al., 2018). Nevertheless, more than 90% of RNA editing occurs in Alu repetitive elements, and almost all of them are found in intronic regions (Picardi et al., 2014), with the potential to affect splicing and other aspects related to the processing of pre-mRNAs and noncoding transcripts as well as the biosynthesis of circular RNA (Wang et al., 2021).

In general, both the expression of ADARs and the levels of A-to-I RNA editing are higher in BC than those in normal tissues (Fumagalli et al., 2015), but when it comes to the roles of lncRNAs in this modification, our knowledge comes mostly from research on other human cancers. Possibly the best studied example is prostate cancer antigen 3 (PCA3), an intronic lncRNA antisense to PRUNE2 a tumor suppressor gene whose downregulation promotes the development of prostate cancer. Salameh et al. demonstrated that lncRNA PCA3 binds to PRUNE2 pre-mRNA and forms a double-stranded RNA, inducing the process of A-to-I editing, which has been associated with decreased cellular levels of PRUNE2 (Salameh et al., 2015).

Importantly, lncRNAs themselves are subject to RNA editing. HIF1A-AS2 (hypoxia inducible factor 1-alpha natural antisense transcript 2) is upregulated in the MCF-7 BC cell line and downregulates its sense partner, HIF-1A, in the early stages of hypoxia, most likely through a cis-acting feedback loop. HIF-1A influences tumor growth by controlling the expression of angiogenesis-related genes. Due to its capability to form double-stranded RNA structures, transcripts of HIF1A-AS2 undergo ADAR1-dependent A-to-I editing, while knockdown of ADAR1 prevents the RNA editing process, which increases the expression of HIF1-AS2, impacting cellular levels of HIF1. Therefore, the findings suggest ADAR1 coordinates the reciprocal regulation of HIF-1A and HIF1A-AS2, but the exact mechanism has yet to be determined (Picardi et al., 2014; Ma et al., 2019). Similarly, expression of LINC00944 is correlated with cellular levels of ADAR1, but in this case it is speculated that it might be mediated by ADAR’s interactions with Dicer or Staufen proteins. Notably, low levels of LINC00944 are associated with poor survival rates in BC patients; hence, the lncRNA has the potential to be used as a BC biomarker (de Santiago et al., 2021).

Deamination of cytidine to uracil (C-to-U) by APOBEC (apolipoprotein B mRNA editing enzyme, catalytic polypeptide-like) deaminases represents another type of RNA editing. During translation, the edited cytidine is read as thymine. Similar to A-to-I editing, this affects protein-coding sequences as well as noncoding sequences, with possible implications ranging from RNA stability to splicing (Asaoka et al., 2019). APOBEC3B (apolipoprotein B editing catalytic subunit 3B) itself is upregulated in BC tumors and BC cell lines, which correlates with increased C-to-U editing. In agreement with this finding, knockdown of APOBEC3B mRNA decreases deaminase activity in BC (Harris, 2015). Available studies show that editing by APOBEC3 enzymes in BC tumors may improve patient survival by increasing immune system activity (Asaoka et al., 2019). Nonetheless, there are no reports on possible roles of lncRNAs mediating or modulating the process in BC, which warrants further research.

### Other Chemical Modifications of LncRNAs

Adenosine methylation at the nitrogen-6 position (referred to as N6-methyladenosine or m6A) represents a widespread, reversible modification of mRNAs and ncRNAs (Linder et al., 2015; Wang et al., 2016; Wang T. et al., 2020) (Figure 3B). It is controlled by three types of enzymes: the one that recognizes the modification (*readers*), methyltransferase complex that catalyzes the process (*writers*), and demethylases that reverse the methylation (*erasers*). It affects virtually all aspects of RNA biology, including RNA transcription, processing and translation (Zhou et al., 2019; Jiang et al., 2021; Liu, 2021). Fat mass and obesity-associated protein (FTO) is one of the demethylases whose knockdown in MDA-MB-231 and MCF-7 BC cell lines leads to suppressed cell growth, colony-forming ability, and increased apoptosis. *In vivo*, FTO is linked to promoting breast tumor growth and metastasis (Niu et al., 2019).

M6A methylation of selected lncRNAs stabilizes them, leading to their heightened cellular levels, as is the case in DLGAP1-AS1 and LINC00958. The former is stabilized by the m6A methyltransferase WT1-associated protein (WTAP), which is linked to a more resistant BC phenotype (Huang et al., 2021). Similarly, upregulation of LINC00958 through m6A methylation promotes BC progression (Rong et al., 2021). In contrast, the cytoplasmic lncRNA LINC00675 acts as a sponge for the microRNA miR-513b-5p, suppressing carcinogenesis. Interestingly, although just one m6A site is found in the LINC00675 transcript, it is required for sponging activity (Fan and Wang, 2021).

LncRNA MALAT1 (metastasis-associated lung adenocarcinoma transcript 1) expression was shown to be upregulated in several types of cancer, including BC, where it can induce migration and stimulate the growth of cancer cells (Schmidt et al., 2011; Barsoum et al., 2020). In BC, MALAT1 undergoes m6A modification mediated by METTL3 methyltransferase. MALAT1 acts as a decoy for miRNA miR-26b, whose expression drops in BC. As miR-26b targets and downregulates HMGA2 (high mobility group AT-hook 2), m6A methylation of MALAT1 indirectly increases HMGA2 expression, with an effect on epithelial-mesenchymal transition (EMT), a mechanism for initiating the invasive and metastatic behavior of epithelial cancers. Notably, high expression of METTL3 and MALAT1 is typically seen in BC patients with poor prognosis (Zhao C. et al., 2021). MALAT1 itself represents one of the most promising candidates for targeted BC therapy, as its knockdown in a mouse model using antisense oligonucleotides results in slower tumor expansion, significant differentiation into cystic tumors, and decreased metastasis (Arun et al., 2016).

Pseudouridilation, also called 5-ribosyuracil, is found across diverse classes of RNA, including lncRNAs (Figure 3C). This isomerization of uracil is conducted by pseudouridine synthases (PUSs) and is likely to be irreversible. Functionally, it does not change the way adenosine binds to uracil—now being a pseudouridine (ψ)—but it affects interactions with other bases. ψ was found in coding and noncoding RNAs and is known to increase the stability of tRNAs and affect the processing of rRNAs (Dinescu et al., 2019; Zhang and Pan, 2022). Li et al. indicated that out of the 195 ψ sites identified by them, as many as 170 were found in lncRNAs frequently associated with disease development, e.g., MALAT1 or XIST, but their functions are not fully understood and require further research (Li et al., 2015). One of the factors involved in pseudouridilation is DKC1 (dyskerin pseudouridine synthase 1), with high expression levels found in a variety of human malignancies and correlated with poor outcome in BC patients. Studies have shown that targeted silencing of DKC1 using RNAi (RNA interference) reduces gene expression in BC cell lines, resulting in decreased pseudouridilation in rRNAs and affecting the survival of proliferating cells (Montanaro et al., 2006; Elsharawy et al., 2020).

### Methods for the Study of LncRNAs in BC Epigenetics

Several features of lncRNAs, such as low endogenous abundance and high tissue specificity, make them challenging for functional analysis, but recent developments in molecular biology and bioinformatics provide improved ways to characterize these RNAs. In particular, a range of high-throughput approaches have been applied to decipher the interplay between lncRNAs and epigenetic machinery in BC. Chromatin isolation by RNA purification (ChIRP), capture hybridization analysis of RNA targets (CHART), and RNA antisense purification (RAP) are RNA-centric approaches that enable genome-wide mapping of lncRNA binding sites (Chu et al., 2011; Simon et al., 2011; Engreitz et al., 2014). In these techniques, biotinylated tiling oligonucleotides and streptavidin magnetic beads are used to retrieve a target lncRNA that is bound to DNA. Of note, while a pool of oligonucleotides that tile to the full length of the RNA are used in ChiRP and RAP, CHART is limited to a couple of short probes. Chu et al. applied ChIRP to map HOTAIR occupancy genome-wide in HOTAIR-expressing MDA-MB-231 BC cells. Using two separate ChIRP probe sets, they discovered 832 HOTAIR occupancy locations across the genome Chu et al. (2011). The same assay was applied to decipher the role of MaTAR25 lncRNA in BC progression (Chang et al., 2020).

In the protein-centric methods, i.e., those concentrated on the study of lncRNA-protein interactions, antibodies are used to immunoprecipitate the endogenous proteins of interest along with their interacting RNA, followed by recovery of the RNA, which is then subjected to sequencing or qPCR. There exist countless variations of this approach, but the main distinction refers to immunoprecipitation, which can either be native or crosslinked. RIP can be applied with or without crosslinking (Gagliardi and Matarazzo, 2016). Using native RIP, Chu et al. found that EZH2 histone methyltransferase suppresses PHACTR2-AS1 expression in BC cells, with PHACTR2-AS1 depletion leading to hyperactivation of ribosome synthesis and ribosomal DNA instability, which promotes cancer cell proliferation and metastasis (Chu et al., 2020). Crosslinking and immunoprecipitation (CLIP) represents another protein-centric technique, with its characteristic feature being the irradiation of cells with UV light to covalently capture close associations between RNAs and proteins (Ule et al., 2003). Kim et al. employed a CLIP assay and demonstrated that TEAD, a prometastatic transcription factor, is bound and inactivated by MALAT1, preventing TEAD from associating with its coactivator YAP and targeting gene promoters (Kim et al., 2018). The aforementioned RAP) technique is sometimes coupled with mass spectrometry (RAP-MS) to additionally identify direct protein interactors for a given RNA molecule (McHugh and Guttman, 2018). For instance, RAP-MS was employed to identify 701 proteins that interact with lincNMR, a lncRNA overexpressed in BC (Gandhi et al., 2020). As a result, Gandhi et al. deciphered a mechanism of action for this tumor-promoting lncRNA, underscoring its regulatory roles in nucleotide metabolism.

It is noteworthy that in contrast to protein-coding genes, the majority of human lncRNAs have no orthologs in mouse. Nevertheless, an emerging body of evidence indicates that some of those non-conserved lncRNAs are functional. One of the major techniques used to study gene function is cell culture. However, cultured cells often exhibits significant deviations from genuine physiological conditions. To address this issue, humanized mouse models (mice that contain functioning human genes, cells, tissues) are implemented. Recently, Ruan et al. developed a liver-specific model, showing that LINC01018 interacts with HuR to perform its function. Surprisingly, the authors were unable to demonstrate the same function in primary hepatocytes, which supports the notion that determining the biological role of non-conserved human lncRNA genes may necessitate the use of an *in vivo* system (Ruan et al., 2020).

### Epigenetics-Oriented Application of LncRNAs in BC Diagnostics and Therapies

There are at least several qualities that make lncRNAs suitable targets for cancer therapies. First, the ability to kill cancer cells selectively is enabled by their specific expression, while because of their low expression, lncRNA-targeting drugs can be used at lower doses, thus mitigating issues associated with their toxicity. Additionally, unlike protein-coding genes participating in cellular signaling pathways that include signal amplification cascades, lncRNAs function at absolute expression levels, making manipulation easier. As a result, lncRNAs have attracted much research attention as potential targets for BC therapy, and an increasing number of lncRNAs have been subject to preclinical studies. A possible approach in the fight against cancer is the suppression of oncogenic lncRNAs, those upregulated in cancer. Their levels can be lowered using various technologies, with a possible effect on epigenetic modifications. One of them is to apply ASOs, single-stranded oligonucleotides that are complementary to the target lncRNAs, forming a DNA/RNA duplex that is then cleaved by RNase H (Lee and Mendell, 2020). ASOs are commonly used to alter mRNA expression and have been successful in treating diseases, such as neurodevelopmental disorders (Hill and Meisler, 2021), but currently only a couple of drugs are approved by the US Food & Drug Administration (FDA) and are available on the market, including EXONDYS 51 against Duchenne muscular dystrophy or SPINRAZA to cure spinal muscular atrophy. ASOs of various designs, such as locked nucleic acid GapmeRs and antagonists to NATs (antagoNATs), are used to provide diverse means to target epigenetic modifications *in vivo* for therapeutic purposes (Sarma et al., 2010; Lennox and Behlke, 2020; Taiana et al., 2021). Nevertheless, extensive pre-clinical research does not translate into clinical applications as there are factors that prevent widespread usage of ASOs in therapeutics (Xia et al., 2019; Zhao et al., 2020). One of the obstacles is the delivery of oligonucleotides specifically to the target organs or cancer cells. The delivery to undesired locations may lead to alteration in gene expression profiles that could be hazardous for healthy tissues. When it comes to lncRNAs, their advantage is that typically they are either (over) expressed in cancer cells or play redundant roles in healthy tissues, thus their knockdown has lower chances for undesired consequences. Other limitations to keep in mind are off-target interactions and toxicity of the used chemistry and there are hopes to mitigate them with recently developed technology, such as endogenous vesicle loading, spherical nucleic acids or nanotechnology solutions (Roberts et al., 2020). There also exist alternatives to ASOs as well, including small interfering RNAs (siRNAs) and short hairpin RNAs (shRNAs), deoxyribozymes, and ribozymes, as well as a range of genome engineering tools, such as transcription activator-like effector nucleases (TALENs) and the clustered regularly interspaced short palindromic repeats (CRISPR)/Cas9 system (Dizaji, 2020).

In addition to targeting the expression of lncRNAs, several technologies have been proposed to disrupt lncRNA/protein interactions, thus disabling their functions related to epigenetic regulation. Small molecules, for instance, bind to lncRNAs or RNA-binding proteins (RBPs), alter their secondary or tertiary structures, or directly mask protein-binding sequences to disrupt interactions (Fatemi et al., 2015; Zhong et al., 2021). Small molecules were used in a BC model to interfere with the interactions of HOTAIR and PRC2 or LSD1 complexes, limiting tumor metastatic potential (Tsai et al., 2011). Another example is PIM serine/threonine kinase, which affects H19 expression in cells by regulating methylation of the H19 promoter (Singh et al., 2020). H19 overexpression has been linked to the progression of tumors. As a result, in clinical trials, the use of pan-PIM inhibitors indirectly regulates the level of H19 in tumor cells to exert an anticancer effect (Singh et al., 2020). siRNAs targeting BC-related lncRNAs (such as HOTAIR) have been shown to inhibit breast cancer growth and invasion (Li Z. et al., 2017). The application of ASOs could also be exemplified by LINC02273, a lncRNA stabilized by hnRNPL. The recruitment of the hnRNPL-LINC02273 complex to the promoter of AGR2 (anterior gradient 2, a disulfide isomerase associated with decreased breast cancer survival) increases local H3K4me3 and H3K27 acetylation, thereby epigenetically upregulating AGR2 and promoting BC metastasis. An ASO targeting LINC02273 inhibits the formation of the hnRNPL-LINC02273 complex, thereby reducing AGR2 expression and inhibiting BC metastasis *in vitro* and *in vivo* (Xiu et al., 2019).

Multiple lncRNAs are known to indicate the presence or progression of human cancers and are referred to as tumor markers. They can be detected in either body fluids or tumor tissues. Biomarkers detected in body fluids (particularly in blood serum) are easily measured, and their diagnostic performance in multiple cancers has been confirmed. The US Food and Drug Administration (FDA) has approved carcinoembryonic antigen (CEA) and cancer antigen 15-3 (CA15-3) in serum as biomarkers for BC. Even though CEA and CA15-3 are widely used in the diagnosis of BC, they have some limitations, most notably low sensitivity and specificity. As a result, it is critical to identify novel molecular markers that would grant an improved diagnostic utility. One possibility is to employ lncRNAs and lncRNA-mediated epigenetic modifications, especially since BC is distinguished by aberrant DNA methylation and other epigenetic alterations. Using high-throughput methylation data from BC and normal breast tissues, Panagopoulou et al. identified 11,176 to 27,786 differentially methylated genes (DMGs) in relation to clinically relevant endpoints (Panagopoulou et al., 2021). These alterations in methylation are also evident in lncRNAs. A recent epigenome-wide association study (EWAS) demonstrated that LINC00299 hypermethylation in the peripheral blood of TNBC patients can be used as a useful circulating biomarker with excellent diagnostic value (Bermejo et al., 2019), while Zhao et al. performed a comparative study of histone modifications at the promoters and enhancers of lncRNAs: lysine H3K27 acetylation (H3K27ac), H3K27me3, trimethylation of Lys36 in histone H3 (H3K36me3), monomethylation of histone H3 at lysine 4 (H3K4me1), and trimethylation of histone H3 at lysine 4 (H3K4me3). Interestingly, it was demonstrated that 92.6% of lncRNAs show subtype-specific epigenetic alterations, with only a small number of common deregulated lncRNAs shared by different subtypes (Zhao H. et al., 2021). They found that epigenetically dysregulated lncRNAs associated with enhancers play an important role in controlling subtype-specific expression and biological functions. They also proposed six lncRNAs with differential histone modifications at enhancers to be used as prognostic biomarkers of the basal subtype of BC (LINC00393, RP1-140K8.5, KB-1836B5.1, CASC11, AC020916.2, and AC005162.1) and three lncRNAs with differential DNA methylation in enhancers or promoters (CTC-303L1.2, SLC26A4-AS1, and RP11-738B7.1) as prognostic biomarkers of the luminal subtype. In the basal subtype, five lncRNAs (AC020916.2, RP1-140K8.5, AC005162.1, CTC-303L1.2, and RP11-738B7.1) were identified as biomarkers. Finally, the active enhancer marker H3K27ac was found to modulate the expression of RP1-140K8.5, AC005162.1, LINC00393, KB-1836B5.1, and AC020916.2, all of which have potential tumor-promoting activities and therefore represent candidates for gene therapy approaches.

Although the above examples collectively confirm the rightness of searching for new lncRNAs as diagnostic and prognostic markers in BC, their detection in blood and biopsies tends to be challenging due to inherent instability and/or low copy number. With significant improvements in throughput and accuracy, RNA-seq has the potential to overcome these limitations. In recent years, Oxford Nanopore Technology (ONT) introduced direct RNA sequencing (DRS), the first approach that enables sequencing of RNA without the need of conversion to cDNA, fragmentation, and amplification (Garalde et al., 2018). Thus, by eliminating these steps and biases associated with them, DRS technology may be useful in detecting lncRNAs with low expression levels and stability. Additionally, because of the short sample processing time and high accuracy, new nanomaterials-based technologies offer alternative methods for detecting cancer biomarkers. Several nanotechnology-based techniques are now being investigated for the detection of lncRNAs (reviewed in Sargazi et al., 2022). Possibly the most popular nanoparticles (NPs) used in cancer research are gold NPs (AuNPs). Recently, Lou et al. combined reverse transcription-coupled loop-mediated isothermal amplification (RT-LAMP) with aggregation of positively-charged gold nanoparticles [(+)AuNPs]. The technique allowed efficient detection of HOTTIP lncRNA in diluted serum, proving its utility in diagnosis of pancreatic ductal carcinoma (Lou et al., 2020).

### Prospects and Challenges

One of the challenges with the clinical application of lncRNAs and epigenetic modifications is determining a simple and quick technique for detecting the target in BC patients. Therefore, lncRNA-based diagnosis and prognosis biomarker studies will need to place a greater emphasis on serum circulating lncRNAs, and one needs to keep in mind that the concentration of targeted lncRNAs may be lower than the detection limit of current equipment. Moreover, as lncRNAs play roles in various processes related to BC development, the mechanism behind deregulation of lncRNAs and associated epigenetic/epitranscriptomic phenomena should be investigated thoroughly, especially because almost all studies on lncRNAs are performed in cell lines, and translation of the results to diverse subtypes of BC is not a straightforward task. It should also be noted that lncRNAs have secondary and tertiary structures, which may render lncRNAs targeting therapeutics ineffective. Finally, a single lncRNA may be insufficient for cancer diagnosis. According to Xie et al., a diagnostic panel for NSCLC (non-small-cell lung carcinoma) has higher specificity (79.2%) and sensitivity (77.1%) than any single molecular marker, such as CEA and lncRNA ANRIL (Xie et al., 2018). Indeed, biomarkers in a panel can complement one another, resulting in better diagnostic performance. There already exist plenty of multigene panels that assist doctors in determining the outcomes of treatment and prognosis. The Oncotype DX test (https://www.oncotypeiq.com, by Genomic Health, Inc.), exclusively applicable to ER-positive tumors, can predict whether BC will recur. It also predicts whether one would benefit from chemotherapy as well as hormone therapy. The MammaPrint® test (https://agendia.com, by Agendia Inc.) examines the 70 most significant genes linked to recurrence of BC, while EndoPredict (https://endopredict.eu/, by Myriad Genetics GmbH) is an RNA-based multigene test that predicts the likelihood of distant recurrence in patients receiving adjuvant endocrine therapy for ER + and HER2 breast cancer. Other tests also exist (Vieira and Schmitt, 2018); however, they heavily rely on the expression of protein-coding genes, completely disregarding the progress made in the field of noncoding RNAs, leaving a space for future improvements. An interesting alternative that opened up new horizons in research on cancer diagnosis are lncRNA-encoded micropeptides. In spite of the fact that only a handful of micropeptides have been characterized, they may serve as excellent diagnostic and prognostic biomarkers thanks to their cancer-type specificity (Polankowski et al., 2022; Setrerrahmane et al., 2022).

Along with gene expression-based tests, there are also commercially available assays devoted to detecting aberrant DNA methylation in BC, such as Therascreen® PITX2 RQG by QIAGEN N.V./Therawis Pharma GmbH. The Therascreen® PITX2 RGQ PCR Kit is a qPCR-based assay that determines the ratio of methylated to unmethylated DNA content in tumor histology sections, where the percent methylation ratio (PMR) is indicative of overall survival and patient outcome when anthracyclines are combined with standard therapy (Maier et al., 2007). Anthracyclines carry serious side effects that may limit treatment (Volkova and Russell, 2012), and patients with less aggressive tumor subtypes or other contraindications may be adequately treated with standard approaches (Turner et al., 2015). By using the Therascreen® PITX2 assay, the risk of overtreatment can be minimized, however the assay is limited to estrogen receptor-positive, node-negative tumors only. The more aggressive and/or difficult-to-treat HER2-positive and triple-negative subtypes or tumors with lymph node involvement do not benefit from this assay. More assays available for different cancers are reviewed elsewhere (Taryma-Leśniak et al., 2020). Nevertheless, protein-coding genes are harnessed there, with no lncRNA-based diagnostic or prognostic test currently commercially available, and lncRNA-related epigenetic and epitranscriptomic modifications are completely neglected. In this light, it is also not surprising that out of 3,777 ongoing clinical trials related to BC, only a couple are linked to lncRNAs (https://clinicaltrials.gov), highlighting the need for additional research, including clinical studies, to benefit from implementing lncRNA markers in clinical practice.
